# Cytotoxic T-Lymphocyte Associated Antigen 4 Polymorphisms and Asthma Risk: A Meta-Analysis

**DOI:** 10.1371/journal.pone.0042062

**Published:** 2012-07-26

**Authors:** Wei Nie, Jiquan Chen, Qingyu Xiu

**Affiliations:** Department of Respiratory Disease, Shanghai Changzheng Hospital, Second Military Medical University, Shanghai, China; Ludwig-Maximilians-University Munich, Germany

## Abstract

**Background:**

A number of studies assessed the association of *cytotoxic T-lymphocyte associated antigen 4* (*CTLA-4*) gene polymorphisms with asthma in different populations. However, the results were contradictory. We performed a meta-analysis to examine the association between *CTLA-4* polymorphisms and asthma susceptibility.

**Methods:**

Pubmed, EMBASE, HuGE Navigator, and Wanfang Database were searched. Data were extracted independently by two reviewers. Odds ratios (ORs) with 95% confidence intervals (CIs) were used to assess the strength of associations.

**Results:**

Seventeen studies involving 6378 cases and 8674 controls were included. Significant association between +49 A/G polymorphism and asthma was observed for AA vs. AG+GG (OR = 1.18, 95% CI 1.01–1.37, *P* = 0.04). There were no significant associations between −318 C/T, −1147 C/T, CT60 A/G, −1722 C/T, or rs926169 polymorphisms and asthma risk.

**Conclusions:**

This meta-analysis suggested that the +49 A/G polymorphism in *CTLA-4* was a risk factor for asthma.

## Introduction

Asthma is a major public health problem worldwide. The disease affects over 300 million people [1]. In developed countries, the prevalence of asthma has increased considerably over the past three decades [2]. Asthma is a complex inflammatory disorder that results from interactions between more than 100 susceptibility genes and multiple environmental factors [3], [4]. It is, therefore, important to identify the gene variants contributing to asthma pathogenesis. Numerous studies have focused on this field, and the cytotoxic T-lymphocyte associated antigen 4 (*CTLA-4*) gene has been extensively studied.

CTLA-4, a B7-binding protein, was initially described as a classical type I glycoprotein on the surface of activated T cells [5]. Cumulative evidence suggested that CTLA-4 may play an important role in the pathogenesis of asthma. CTLA-4 is a powerful negative regulator of T cell activation and is associated with Th cell differentiation. Oosterwegel et al. [6] demonstrated that CTLA-4 was a potent and critical inhibitor of Th2 cell differentiation. Expression of CTLA-4 in Th2 cells was much higher than in Th1 cells [7]. CTLA-4 was also demonstrated to suppress the production of cytokines produced by Th2 cells [7]. A number of studies showed that administration of CTLA-4-Ig significantly ameliorated airway hyperresponsiveness (AHR), reduced the level of eosinophils in average bronchoalveolar lavage fluid and serum IgE, as well as cytokine production in murine asthma model [8]–[11]. Recently, Choi et al. [12] reported that intranasal administration of Hph-1-ctCTLA-4 could significantly reduce infiltration of inflammatory cells, secretion of Th2 cytokines, serum IgE levels and AHR in a mouse model of allergic airway inflammation. Lin and co-workers demonstrated that decreased allergic inflammation by surfactant protein D was mediated by an increased expression of CTLA-4 in T cells [13].

The human *CTLA-4* gene is located on chromosome 2q33.2 [14]. Several single nucleotide polymorphisms (SNPs) of the *CTLA-4* gene have been identified. Some of these studies have demonstrated a significant association of *CTLA-4* polymorphisms with atopy or asthma [15]–[17]. However, the results were not consistent in other studies [18], [19].

Considering a single study may lack the power of providing a reliable conclusion, we performed a meta-analysis to investigate the relationship between *CTLA-4* gene variants and asthma. To our knowledge, this is the first meta-analysis of the association between *CTLA-4* polymorphisms and asthma susceptibility.

## Methods

### Publication search

Pubmed, EMBASE, HuGE Navigator, and Wanfang Database were searched (Last search was updated on March, 2012). The following MeSH terms were used in Pubmed: “asthma” and “polymorphism, genetic” and “CTLA 4 antigen”. The search terms used in EMBASE and Wanfang Database were as follows: (asthma or asthmatic) and (cytotoxic T-lymphocyte associated antigen 4 or CTLA4 or CTLA-4 or CD152) and (polymorphism or mutation or variant). We also searched the reference list of original reports and review articles related to *CTLA-4* polymorphism and asthma risk to identify studies not included in the computerized databases.

### Inclusion and exclusion criteria

Studies fulfilled the following criteria were included in this meta-analysis: (1) asthma diagnosed by a physician or according to asthma guidelines, (2) evaluation of the polymorphisms in *CTLA-4* gene and asthma risk performed, (3) using a case-control design, (4) genotype distributions in both asthma cases and controls should be available for estimating an odds ratio (OR) with 95% confidence interval (CI). Studies were excluded if one of the following existed: (1) not clinical studies, and (2) reviews and abstracts. For overlapping studies, only the one with the largest sample size was included. There was no language restriction.

### Qualitative assessment

Two authors independently assessed the quality of each study. Any disagreement was resolved by consensus. Quality assessment scores of molecular association studies of asthma were used to assess the quality of selected articles [20]. This quality scoring system was based on both traditional epidemiologic considerations and genetic issues. Total scores ranged from 0 (worst) to 15 (best). Studies with quality scores ≤4 were defined as low quality studies [21].

### Data extraction

Two investigators (Nie and Chen) independently reviewed full manuscripts of eligible studies, and the relevant data were extracted into predesigned data collection forms. We verified accuracy of data by comparing collection forms from each investigator. Any discrepancy was resolved by discussion or a third author (Xiu) would assess these articles. The following data were collected from each study: first author's name, year of publication, original country, ethnicity, age, atopic status,sample size, asthma and atopy definition, genotyping method, the polymorphisms in *CTLA-4* gene, and genotype number in cases and controls. Authors of the included studies were contacted via E-mail when additional study data were needed.

### Statistical analysis

When the data from at least 2 similar studies were available, meta-analysis was performed. ORs and 95% CIs were employed to assess the strength of association between SNPs in +49 A/G, −318 C/T, −1147 C/T, CT60 A/G, −1722 C/T, rs926169 and asthma risk. OR1, OR2, and OR3 were calculated for the genotypes 1) AA vs. GG (OR1), AG vs. GG (OR2), and AA vs. AG (OR3) for the +49 A/G and CT60 A/G polymorphisms, 2) CC vs. TT (OR1), CT vs. TT (OR2), and CC vs. CT (OR3) for the −318 C/T, −1147 C/T, and −1722 C/T polymorphisms, and 3) AA vs. CC (OR1), AC vs. CC (OR2), and AA vs. AC (OR3) for the rs926169, respectively. These pairwise differences were used to indicate the most appropriate genetic model as following: if OR1 = OR3≠1 and OR2 = 1, then a recessive model was suggested; if OR1 = OR2≠1 and OR3 = 1, then a dominant model was suggested; if OR2 = 1/OR3≠1 and OR1 = 1, then a complete overdominant model was suggested; if OR1>OR2>1 and OR1>OR3>1 (or OR1<OR2<1 and OR1<OR3<1), then a codominant model was suggested [22]. Once the best genetic model was identified, this model was used to collapse the three genotypes into two groups (except in the case of a codominant model) and to pool the results again. A random-effects model, using the Mantel-Haenszel method, was used to calculate the pooled ORs. The statistical significance of OR was determined with *Z* test.

Departure from Hardy-Weinberg equilibrium (HWE) in controls was tested by the chi-square test. The Q statistic and the *I* ^2^ statistic were used to test for heterogeneity among the studies included in the meta-analysis. Sensitivity analyses were performed by including studies not in HWE. In addition, sensitivity analyses were also done by ethnicity and atopic status. Graphic exploration with funnel plots was used to evaluate the publication bias visually. The Begg's test and the Egger's test were used to assess publication bias statistically [23], [24].

All statistical tests were performed by using the Revman 5.1 software (Nordic Cochrane Center, Copenhagen, Denmark), STATA 11.0 software (Stata Corporation, College Station, TX), and SPSS 18.0 software (Chicago, IL, USA). A *P* value<0.05 was considered statistically significant, except for tests of heterogeneity where a level of 0.10 was used.

## Results

### Literature search and study characteristics

**Figure 1:**
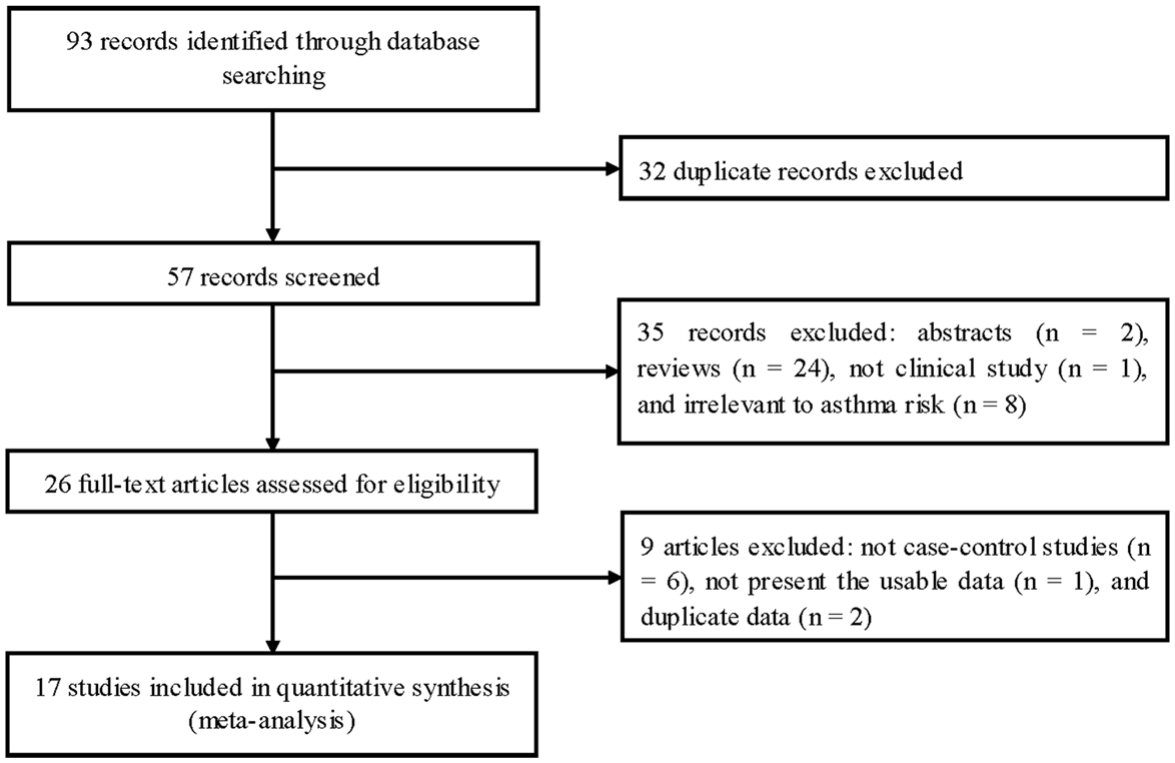
Flow of study identification, inclusion, and exclusion.

**Figure 1** outlines our selection process. Briefly, a total of 93 articles were identified after an initial search. After removing duplications, 32 articles were excluded. After reviewing the titles and abstracts, 35 articles were excluded because of abstracts, reviews, not clinical studies, or irrelevance of asthma risk. After reviewing full texts of the remaining 26 articles, 9 articles were further excluded. One article reported two cohorts [34], and each cohort was considered as a separate case-control study. Finally, a total of 18 case-control studies in 17 articles were identified [15]–[17], [25]–[38], including 6378 cases and 8674 controls. There were 11 studies on +49 A/G, 12 studies on −318 C/T, 6 studies on −1147 C/T, 5 studies on CT60 A/G, 3 studies on −1722 C/T and rs926169. There were 10 studies of Asians [15], [17], [25]–[27], [29], [32], [34], [37], [38] and 6 studies of Caucasians [16], [28], [30], [31], [33], [36]. Five studies were performed in adults [15]–[17], [34], [38], 11 studies in children [25]–[27], [29]–[32], [34]–[37]. Two studies included both adults and juveniles [28], [33]. Five studies included only atopic asthma patients [27], [28], [34], [35], [38]. Four studies included both atopic and non-atopic asthma patients but the data for these patients could be separately extracted 17,25,26,30. Seven studies did not offer detailed information [15], [16], [29], [31]–[33], [36]. Asthma was defined with different criteria (physician's diagnosis, ATS diagnosis criteria, NHLBI/WHO guideline, NIH criteria, and Chinese asthma diagnosis criteria for children). Atopy was defined based on total IgE in 6 studies [16], [17], [25], [27], [28], [32], radioallergosorbent test (RAST) in 2 studies [15], [27], skin prick test to common aeroallergens (SPT) in 9 studies [16], [17], [25], [26], [28], [30], [33], [34], [38], and allergen-specific IgE in 4 studies [26], [29], [30], [37]. The quality scores ranged from 5 to 12, suggesting high quality. The characteristics of each study included in this meta-analysis are presented in **Table 1**. Genotype frequencies and HWE examination results are listed in **Table 2**.

**Table 1:** Characteristics of the case-control studies included in meta-analysis.

First authors/				Age	Atopic	Case	Control	Asthma	Atopy	Quality	Genotyping	*CTLA-4*
references	Year	Country	Ethnicity	group	status	(n)	(n)	definition	definition	score	method	polymorphisms
Nakao [27]	2000	Japan	Asian	Children	Atopic	120	200	Physician's diagnosed	total IgE, RAST	5	PCR-RFLP	+49 A/G, −318 C/T
Hizawa [15]	2001	Japan	Asian	Adults	NA	339	305	ATS diagnosis criteria	RAST	12	PCR-RFLP	+49 A/G, −318 C/T
Howard [16]	2002	Netherlands	Caucasian	Adults	NA	200	201	A algorithm based on	SPT, total IgE	11	PCR-RFLP	−1147 C/T, −658 C/T,
								ATS diagnosis criteria				−318 C/T, +49 A/G
Lee [17]	2002	Korea	Asian	Adults	Mixed*	88	86	ATS diagnosis criteria	SPT, total IgE	11	PCR-RFLP	+49 A/G, −318 C/T
Schubert [31]	2006	Germany	Caucasian	Children	NA	231	270	Physician's diagnosed	NA	9	PCR-RFLP	+49 A/G, −318 C/T
Jasek [28]	2006	Poland	Caucasian	Adults/	Atopic	219	102	NHLBI/WHO guideline	SPT, total IgE	11	PCR-RFLP	−1147 C/T, +49 A/G,
				juveniles								−318 C/T
Qian [32]	2007	China	Asian	Children	NA	90	100	Chinese asthma diagnosis	Total IgE	7	PCR-RFLP	−318 C/T
								criteria for children				
Sohn [25]	2007	Korea	Asian	Children	Mixed*	326	254	Physician's diagnosed	SPT, total IgE	11	PCR-RFLP	+49 A/G, −318 C/T
Chan [29]	2008	China	Asian	Children	NA	298	175	ATS diagnosis criteria	Allergen-specific IgE	9	PCR-RFLP	−1147 C/T, +49 A/G, JO30,
												CT60 A/G, JO31, JO27_1
Daley [33]	2009	Australia	Caucasian	Adults/	NA	644	751	A positive answer to the	SPT	9	Illumina	+49 A/G, −318 C/T,
				juveniles				question: “Has a doctor			Bead Array	−1147 C/T, CT60 A/G,
								ever told you that you had			System	−1722C/T, rs926169,
								asthma?”				rs231731
Berce [30]	2010	Slovenia	Caucasian	Children	Mixed*	102	84	ATS diagnosis criteria	Allergen-specific IgE, SPT	7	PCR-RFLP	CT60 A/G
Oh [26]	2010	Korea	Asian	Children	Mixed*	742	238	ATS diagnosis criteria	Allergen-specific IgE, SPT	11	PCR-RFLP	+49 A/G
Undarmaa 1 [34]	2010	Japan	Asian	Children	Atopic	325	336	NIH criteria	SPT	9	TaqMan-ASA	+49 A/G, −318 C/T
Undarmaa 2 [34]	2010	Japan	Asian	Adults	Atopic	367	676	ATS diagnosis criteria	SPT	9	TaqMan-ASA	+49 A/G, −318 C/T
DeWan [35]	2010	USA	Mixed	Children	Atopic	66	42	Physician's diagnosed	NA	11	Affymetrix	−318 C/T, −1147 C/T, CT60 A/G
											Genome-Wide Human	
											SNP Array 5.0	
Sleiman [36]	2010	USA	Caucasian	Children	NA	793	1988	Physician's diagnosed	NA	12	Illumina HH550 BeadChip	−1722C/T, rs926169
Noguchi [37]	2011	Japan	Asian	Children	Mixed	938	2376	Physician's diagnosed on	Allergen-specific IgE	12	Illumina HumanHap550v3	−1722C/T, rs926169
								the basis of the NIH criteria			/610-Quad Genotyping BeadChip	
Anantharaman [38]	2011	Singapore	Asian	Adults	Atopic	490	490	A positive answer to the	SPT	12	Illumina BeadXpress platform	−318 C/T, −1147 C/T, CT60 A/G
								question: “Have you ever had			and Sequenom platform	
								asthma?” and a doctor's				
								diagnosis				

* Data for atopic or non-atopic asthma patients could be separately extracted.

ATS, American Thoracic Society; NHLBI, The National Heart, Lung, and Blood Institute; WHO, The World Health Organization; NIH, National Institutes of Health; SPT, skin prick test to common aeroallergens; RAST, radioallergosorbent test; PCR, polymerase chain reaction; RFLP, restriction fragment length polymorphism; TaqMan-ASA, TaqMan allele-specific amplification method; NA, not available.

**Table 2:** Distribution of *CTLA-4* genotype among patients and controls included in the meta-analysis.

Studies		Asthma			Control		HWE
	11^a^	12^b^	22^c^	11	12	22	(*P* value)
+49 A/G							
Nakao	27	52	41	32	107	61	0.189
Hizawa	40	178	121	40	140	125	0.935
Howard	76	82	19	39	72	23	0.297
Lee	15	24	49	8	29	49	0.238
Schubert	98	105	28	105	127	38	0.968
Jasek	66	101	52	33	48	21	0.645
Sohn	45	125	156	19	103	132	0.859
Chan	40	119	113	21	75	75	0.737
Daley	238	290	88	291	338	98	0.992
Oh	61	312	369	16	107	115	0.178
Undarmaa 1	49	153	123	43	155	138	0.959
Undarmaa 2	58	175	134	106	323	247	0.981
−318 C/T							
Nakao	97	19	4	157	43	0	0.088
Hizawa	265	71	3	238	65	2	0.278
Howard	144	30	2	115	14	2	0.059
Lee	70	16	2	67	15	4	0.022
Jasek	172	44	3	79	22	1	0.694
Schubert	181	47	3	214	53	3	0.889
Qian	75	13	2	84	15	1	0.721
Sohn	247	77	2	199	54	1	0.182
Daley	537	100	5	616	128	7	0.902
Undarmaa 1	253	67	5	263	68	5	0.801
Undarmaa 2	284	78	5	512	153	11	0.911
DeWan	58	7	1	35	6	1	0.267
Anantharaman	350	128	12	343	134	13	0.799
−1147 C/T							
Howard	108	46	2	97	18	2	0.295
Jasek	146	65	8	66	31	5	0.587
Chan	216	68	7	129	43	3	0.787
Daley	445	181	18	500	226	25	0.931
DeWan	52	13	1	29	12	1	0.853
Anantharaman	335	140	15	307	162	21	0.949
CT60 A/G							
Chan	13	97	183	6	58	109	0.611
Berce	14	62	26	21	34	29	0.093
Daley	191	317	132	229	369	148	0.977
DeWan	8	31	27	4	19	19	0.810
Anantharaman	36	193	261	21	161	308	0.994
−1722 C/T							
Daley	551	89	4	644	102	4	0.986
Sleiman	663	127	6	1666	308	14	0.954
Noguchi	340	463	135	862	1131	391	0.537
rs926169 A/C							
Daley	236	307	100	282	356	111	0.937
Sleiman	284	382	127	743	945	300	0.987
Noguchi	135	455	342	336	1110	938	0.793

a AA or CC;

b AG, CT, or AC;

c GG, TT or CC.

HWE, Hardy-Weinberg equilibrium.

### Quantitative data synthesis

#### The *CTLA-4* +49 A/G polymorphism

**Figure 2:**
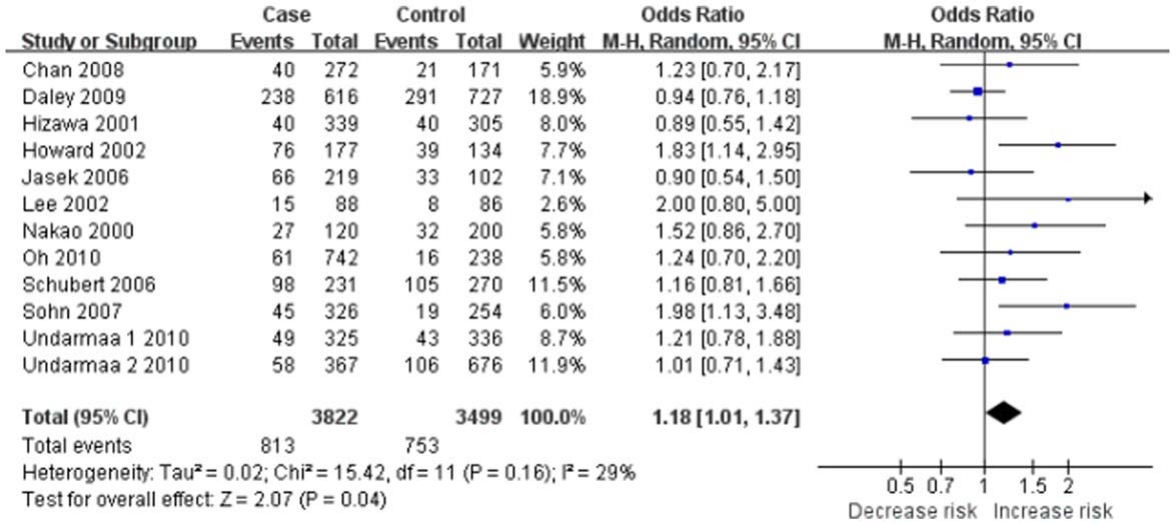
Meta-analysis with a random-effects model for the association between asthma risk and the *CTLA-4* +49 A/G polymorphism (AA vs. AG+GG).

**Figure 3:**
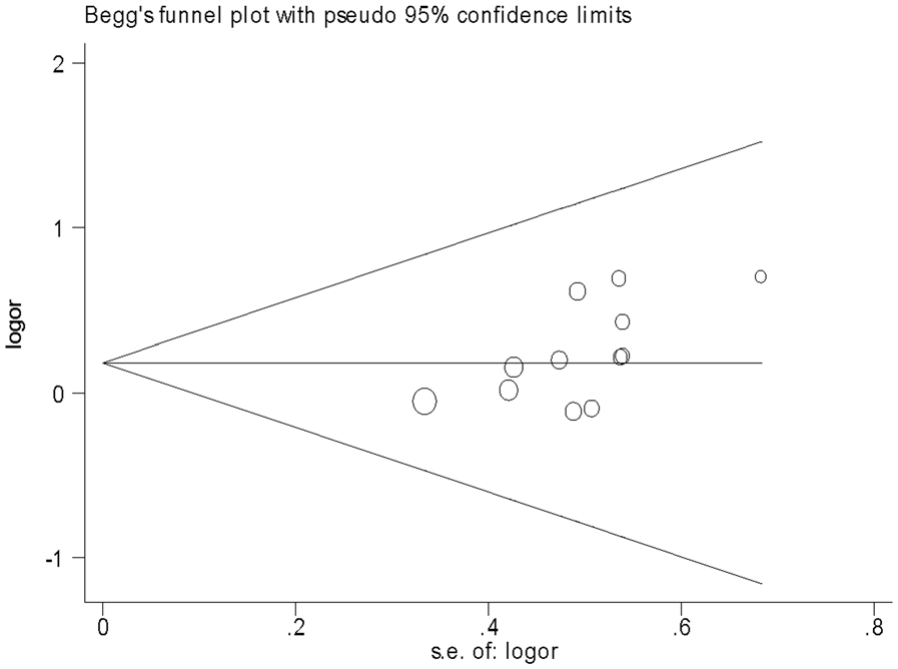
Funnel plot for publication bias in selection of studies on the *CTLA-4* +49 A/G polymorphism (AA vs. AG+GG).

Eleven studies determined the association between +49 A/G polymorphism and asthma [15]–[17], [25]–[29], [31], [33], [34]. Total sample sizes for asthma and control groups were 3822 and 3499, respectively. All studies in HWE were included in pooling. The estimated OR1, OR2 and OR3 were 1.18, 1.02, and 1.16, respectively (**Table 3**). These estimates suggested a recessive genetic model, therefore AG and GG were combined and compared with AA. The pooled OR was 1.18 (95% CI 1.01–1.37, *P* = 0.04) (**Figure 2**). The exclusion of studies with Asians altered the significance of the result (OR = 1.12, 95% CI 0.85–1.48, *P* = 0.41). However, the exclusion of studies with Caucasians did not change the result (OR = 1.21, 95% CI 1.01–1.46, *P* = 0.04). Sensitivity analyses were also performed by atopic status. Borderline yet significant increase of asthma risk was found among the AA carriers of atopic asthma patients (OR 1.26, 95% CI 1.00–1.59, *P* = 0.05). The funnel plot was slightly asymmetrical (**Figure 3**). Begg's test and Egger's test indicated significant publication bias (*P* = 0.016 and *P* = 0.025, respectively).

**Table 3:** Determination of the genetic effects of *CTLA-4* polymorphisms on asthma and sensitivity analyses.

Polymorphisms	Study	Sample size		No. of	Test of association			Model	Heterogeneity		
		case	control	studies	OR (95% CI)	*Z*	*P* Value		*χ* ^2^	*P* Value	*I* ^2^ (%)
+49 A/G											
AA vs. GG	Overall	2108	1875	12	1.18 (1.00–1.40)	1.98	0.05	R	12.78	0.31	14.0
AG vs. GG	Overall	3008	2746	12	1.02 (0.91–1.14)	0.34	0.74	R	6.58	0.83	0.0
AA vs. AG	Overall	2529	2377	12	1.16 (0.99–1.36)	1.89	0.06	R	14.76	0.19	25.0
AA vs. AG+GG	Overall	3822	3499	12	1.18 (1.01–1.37)	2.07	0.04	R	15.42	0.16	29.0
AA vs. AG+GG	Asian	2579	2266	8	1.21 (1.01–1.46)	2.01	0.04	R	7.52	0.38	7.0
AA vs. AG+GG	Caucasian	1243	1233	4	1.12 (0.85–1.48)	0.83	0.41	R	6.77	0.08	56.0
AA vs. AG+GG	Atopic	1954	1892	7	1.26 (1.00–1.59)	1.94	0.05	R	8.30	0.22	28.0
−318 C/T											
CC vs. TT	Overall	2710	2903	12	0.99 (0.65–1.51)	0.04	0.97	R	4.62	0.95	0.0
CT vs. TT	Overall	699	798	12	0.99 (0.64–1.52)	0.04	0.97	R	5.90	0.88	0.0
CC vs. CT	Overall	3344	3610	12	1.03 (0.92–1.16)	0.52	0.61	R	5.38	0.91	0.0
CC+CT vs. TT	Overall	3391	3657	12	0.96 (0.63–1.47)	0.18	0.86	R	4.64	0.95	0.0
CC+CT vs. TT	HWE	3479	3743	13	1.01 (0.67–1.52)	0.03	0.98	R	5.38	0.94	0.0
CC+CT vs. TT	Asian	2057	2361	7	0.91 (0.55–1.51)	0.36	0.72	R	4.16	0.66	0.0
CC+CT vs. TT	Caucasian	1268	1254	4	1.06 (0.48–2.34)	0.14	0.89	R	0.29	0.96	0.0
CC+CT vs. TT	Atopic	1793	2058	6	0.97 (0.57–1.65)	0.11	0.91	R	3.70	0.59	0.0
−1147 C/T											
CC vs. TT	Overall	1353	1185	6	1.29 (0.87–1.91)	1.26	0.21	R	1.05	0.96	0.0
CT vs. TT	Overall	564	549	6	1.16 (0.77–1.73)	0.70	0.48	R	1.21	0.94	0.0
CC vs. CT	Overall	1815	1620	6	1.04 (0.81–1.34)	0.31	0.75	R	10.67	0.06	53.0
CT60 A/G											
AA vs. GG	Overall	891	894	5	1.18 (0.80–1.74)	0.82	0.41	R	6.63	0.16	40.0
AG vs. GG	Overall	1329	1254	5	1.20 (0.94–1.52)	1.45	0.15	R	7.01	0.14	43.0
AA vs. AG	Overall	962	922	5	0.95 (0.63–1.45)	0.23	0.82	R	7.95	0.09	50.0
AA+AG vs. GG	Overall	1591	1535	5	1.19 (0.94–1.49)	1.47	0.14	R	6.86	0.14	42.0
−1722 C/T											
CC vs. TT	Overall	1699	3581	3	1.12 (0.90–1.40)	1.01	0.31	R	0.32	0.85	0.0
CT vs. TT	Overall	824	1950	3	1.17 (0.94–1.45)	1.39	0.16	R	0.33	0.85	0.0
CC vs. CT	Overall	2233	4713	3	0.97 (0.86–1.09)	0.54	0.59	R	0.01	0.99	0.0
CC+CT vs. TT	Overall	2378	5122	3	1.15 (0.93–1.41)	1.32	0.19	R	0.37	0.83	0.0
rs926169											
AA vs. CC	Overall	1244	2710	3	0.99 (0.85–1.15)	0.18	0.86	R	1.48	0.48	0.0
AC vs. CC	Overall	1713	3760	3	1.05 (0.93–1.19)	0.74	0.46	R	1.61	0.45	0.0
AA vs. AC	Overall	1799	3772	3	0.96 (0.85–1.09)	0.63	0.53	R	0.07	0.97	0.0
AA+AC vs. CC	Overall	2368	5121	3	1.03 (0.91–1.17)	0.50	0.61	R	2.15	0.34	7.0

vs., versus; R, random-effects model.

#### The *CTLA-4* −318 C/T polymorphism

**Figure 4:**
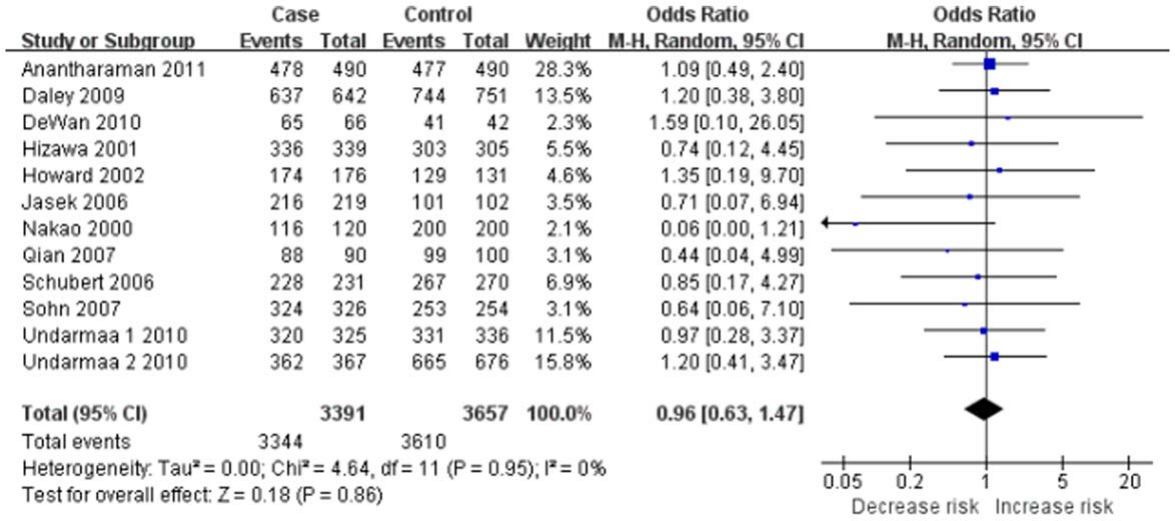
Meta-analysis with a random-effects model for the association between asthma risk and the *CTLA-4* −318 C/T polymorphism (CC+CT vs. TT).

**Figure 5:**
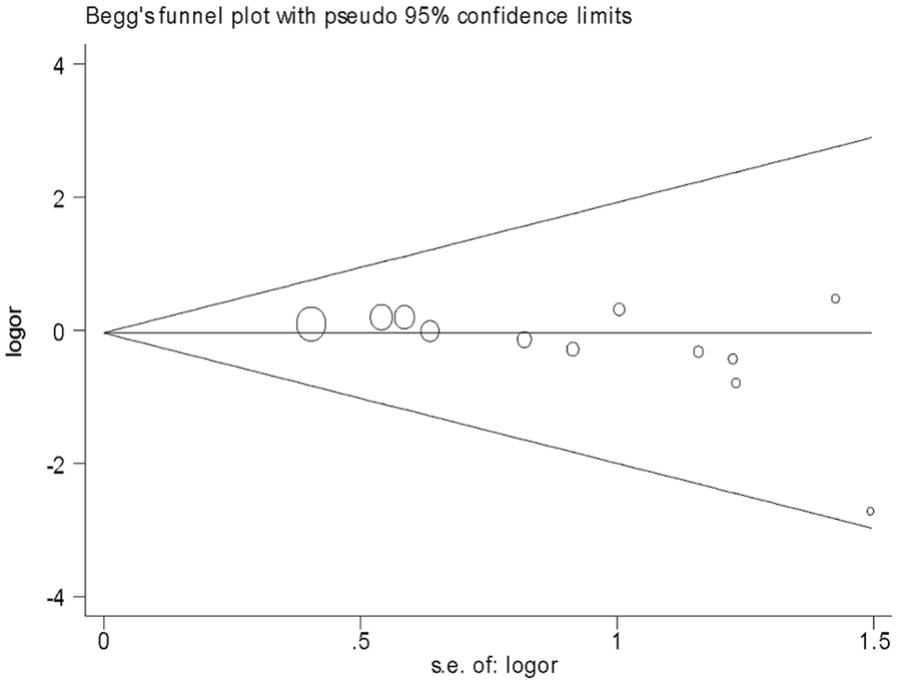
Funnel plot for publication bias in selection of studies on the *CTLA-4* −318 C/T polymorphism (CC+CT vs. TT).

Twelve case-control studies identified an association between *CTLA-4* −318 C/T polymorphism and asthma risk [15]–[17], [25], [27], [28], [31]–[35], [38]. Total sample sizes for asthma and control groups were 3391 and 3657, respectively. All studies in HWE except one study [17] were included in pooling. The estimated OR1, OR2 and OR3 were 0.99, 0.99, and 1.03, respectively (**Table 3**). These estimates suggested a dominant genetic model, therefore CT and CC were combined and compared with TT. The pooled OR was 0.96 (95% CI 0.63–1.47, *P* = 0.86) (**Figure 4**). Sensitivity analysis was performed by including the study [17] that did not observe HWE. The results were similar in showing no genetic effect (OR = 1.01, 95% CI 0.67–1.52, *P* = 0.98). Furthermore, no statistically significant results were found in sensitivity analyses conducted by ethnicity and atopic status (**Table 3**). The funnel plot was slightly asymmetrical (**Figure 5**). Begg's test and Egger's test indicated significant publication bias (*P* = 0.011 and *P* = 0.049, respectively).

#### The −1147 C/T, CT60 A/G, −1722 C/T, and rs926169 polymorphisms

Six studies studied the association between −1147 C/T polymorphism and asthma risk [16], [28], [29], [33], [35], [38]. Total sample sizes for asthma and control groups were 1866 and 1677, respectively. The estimated OR1, OR2 and OR3 were 1.29, 1.16 and 1.04, respectively (**Table 3**). These estimates suggested a codominant genetic model. The pooled OR was 1.29 (95% CI 0.87–1.91, *P* = 0.21) and 1.16 (95% CI 0.77–1.73, *P* = 0.48). Only 5 studies and 3 studies were eligible for meta-analysis on CT60 A/G, −1722 C/T, and rs926169 polymorphisms. Dominant genetic models were chosen based on the estimated OR1, OR2 and OR3 of these three polymorphisms. Results from our meta-analysis demonstrated that CT60 A/G, −1722 C/T, and rs926169 polymorphisms were not risk factors for asthma. Summary of comparisons are listed in **Table 3**.

## Discussion

This meta-analysis of 17 case-control studies including 6378 cases and 8674 controls systematically evaluated the association between +49 A/G, −318 C/T, −1147 C/T, CT60 A/G, −1722 C/T, and rs926169 polymorphisms in the *CTLA-4* gene and asthma risk. We found that +49 A/G polymorphism was a modest risk factor for developing asthma in the overall study population. The results revealed that carriers of the AA homozygote had 18% increased asthma risk compared to those individuals with the G allele carriers (AG+GG). In the sensitivity analysis, we found that individuals carrying AA homozygote had increased asthma risk in Asians, but not in Caucasians. These results suggested that interactions between different ethnicities and genetic variants may contribute to asthma risk. However, there were only 4 studies on Caucasians for this polymorphism [16], [28], [31], [33]. It is therefore possible that the observed ethnic difference was due to chance. More studies with Caucasian population are required to validate the effect of ethnic differences on asthma risk through the +49 A/G polymorphism. In addition, significant heterogeneity was observed in the Caucasians subgroup (*I* ^2^ = 56%) but not in the Asians subgroup (*I* ^2^ = 7%). Furthermore, asthma is a complex disease. Both genetic and environmental factors affect the risk of asthma in different populations. It is possible that different asthma risks in Asians and Caucasians were due to exposure to various environmental factors. However, no reported article was performed to assess the effect of environment-*CTLA-4* interactions in different ethnicities. In the future, more studies should be designed to analyze these associations. We also carried out sensitivity analysis for atopic status. We found that atopic patients had increased asthma risk, suggesting a possibility of atopic status differences in asthma pathogenesis.

Ligers and co-workers showed that CTLA-4 cell-surface expression was significantly increased in individuals carrying the AA genotype, compared to levels in carriers of the AG and GG genotypes [39]. CTLA-4 +49 G>A caused ^17^Ala>^17^Thr substitution in the leading peptide of CTLA-4 [40]. ^17^Thr substitution increased binding of CTLA-4 to B7.1, causing stronger inhibition on T cell activation then CTLA-4 ^17^Ala [41]. In addition, T cells with +49 GG genotype had higher activation and proliferation rates compared to those with +49 AA genotype [41]. Recently, the G allele of the +49 A/G polymorphism was reported to have a strong association with autoimmune diseases [42]–[44]. Considering the inverse relationship between allergic diseases (Th2 dominant) and autoimmune diseases (Th1 dominant), and the role of *CTLA-4* polymorphisms in determining the Th1/Th2 balance [45], it is biologically plausible that the A allele of the +49 A/G polymorphism could increase the susceptibility of asthma. Our findings and a previous study by Yang et al. [46] supported this speculation. Furthermore, Jones et al. [47] indicated +49 A allele was associated with infant atopic dermatitis. However, how A allele of the +49 A/G polymorphism influences asthma risk is unclear. Park et al. [48] reported significantly lower serum sCTLA-4 levels in Behcet's disease patients with the CTLA-4 +49 G allele than those in healthy controls. Serum sCTLA-4 concentrations also increased in patients with allergic asthma and after allergen inhalation in sensitized asthmatic subjects [49]–[52]. These data suggested that +49 A/G polymorphism could influence asthma susceptibility through affecting serum sCTLA-4 level.

Results from our meta-analysis showed the lack of associations between the −318 C/T, −1147 C/T, CT60 A/G, −1722 C/T, or rs926169 polymorphisms and asthma risk. However, these results should be interpreted with caution. Because −318 C/T was shown to be associated with asthma severity and may serve as a clinically useful marker of severe asthma [17]. More studies are required to assess the associations between −1147 C/T, CT60 A/G, −1722 C/T, or rs926169 polymorphisms and asthma risk, since less than 6 case-control studies were included in this meta-analysis. A positive association between these polymorphisms and asthma could not be ruled out because studies with small sample size may have insufficient statistical power to detect a slight effect.

Publication bias and heterogeneity may influence the results of meta-analyses. In our meta-analysis, only studies indexed by the selected databases were included. Negative studies were less likely to be published in journals and be available in computerized database [53], resulting in potential overestimation of effect sizes. In this meta-analysis, Begg's test and Egger's test showed significant publication bias, thus the current results should be interpreted cautiously. In addition, there was no significant heterogeneity in most of the overall comparisons for all 4 polymorphisms. Therefore, heterogeneity did not seem to have influenced the results, suggesting the reliability of our results.

Some limitations of this meta-analysis should be considered. First, the number of available studies that could be included was moderate. Therefore, the results could be influenced by factors like random error. Second, only 7 of the 17 studies were conducted in non-Asian population. Third, the overall outcomes were based on individual unadjusted ORs, while a more precise evaluation should be adjusted by other potentially suspected factors including age, sex and lifestyle. Finally, this study could not address gene-gene and gene-environment interactions, due to insufficient information from the primary publication.

In conclusion, our meta-analysis suggested that the +49 A/G polymorphism in *CTLA-4*, but not the −318 C/T, −1147 C/T, CT60 A/G, −1722 C/T, or rs926169 polymorphisms, represented a risk factor for asthma. Future large-scale studies are still needed to validate our findings. Moreover, gene-gene and gene-environment interactions should also be considered in future studies.
